# Nutritional genomics and metabolomics in obesity and type 2 diabetes

**DOI:** 10.1186/1471-2164-15-S2-O10

**Published:** 2014-04-02

**Authors:** Frank B Hu, Kothandaraman Narasimhan

**Affiliations:** 1 Departments of Nutrition and Epidemiology, Harvard School of Public Health, Boston, MA, USA; 2 Center of Excellence in Genomic Medicine Research, King Abdulaziz University, Jeddah, KSA

## 

Obesity and type-2 diabetes arise from the complex interplay of both genetic and environmental factors. In the past several years, we have seen considerable progress in the use of genomics and metabolomics in investigating the role of diet and nutrition in the etiology of obesity and diabetes. Recent work suggests that the adverse effect of several obesity loci especially FTO may be modified by dietary factors. In a recent study (Qi et al. NEJM 2012), we found a significant interaction between consumption of Sugar-Sweetened Beverages (SSBs) and genetic variants related to Body Mass Index (BMI) in three cohort studies. A genetic-predisposition score was calculated on the basis of 32 BMI-associated loci. We found that the genetic association with BMI was more pronounced among participants with higher intake of SSBs than among those with lower intake. In the combined cohorts, the increases in BMI per increment of 10 risk alleles were 1.00 unit for an intake of < 1 serving/month, 1.12 for 1-4 servings/month, 1.38 for 2-6 servings/week, and 1.78 for ≥ 1 servings/day (P<0.001 for interaction). This analysis suggests that the genetic association with adiposity is amplified by greater intakes of SSBs. In the area of nutrition and metabolomics, several recent epidemiological studies have found that plasma concentrations of metabolites especially Branched-Chain Amino Acids (BCAAs) are associated with increased risk of type-2 diabetes. Other novel metabolite classes have also been linked to diabetes, such as short- and medium-chain acylcarnitines, the specific lipid classes of sphingomyelins (SMs), lysophosphatidylcholines, phosphatidylcholines (PCs), and lysophosphatidylethanolamines. Metabolomics has also been used to characterize the complex human metabolic effects of specific foods, nutrients, and dietary patterns in both intervention and epidemiological studies. Our preliminary analysis found that urinary levels of gut flora metabolites derived from dietary intakes of polyphenols were significantly associated with risk of diabetes.

In summary, both nutrigenomics and metabolomics studies have provided new insights to the etiology of obesity and diabetes and individual differences in response to diet. Continued technological advances in sensitive high-throughput methods and enhanced bioinformatics and analytical tools in combination with carefully conducted population-based studies will enable more widespread use of these technologies in nutrition and metabolic disease research, which will eventually help to achieve the goal of personalized nutrition for prevention and treatment of chronic diseases.

